# Synthesis and structure of 2-amino-4-methylpyridin-1-ium hydrogen squarate

**DOI:** 10.1107/S205698902501045X

**Published:** 2025-11-28

**Authors:** Vanitha Vetrivel, Thangavelu Balakrishnan, Nishandhini Marimuthu

**Affiliations:** a Crystal Growth & Thin Film Laboratory PG& Research Department of Physics Thanthai Periyar Government Arts and Science College (Autonomous) Affiliated to Bharathidasan University, Tiruchirappalli-620 023 Tamil Nadu India; b Department of Bioinformatics Vels Institute of Science, Technology & Advanced Studies,Chennai-600117 Tamil Nadu India; University of Aberdeen, United Kingdom

**Keywords:** crystal structure, hydrogen squarate anion, 2-amino-4-methyl­pyridin-1-ium cation

## Abstract

The title salt (systematic name: 2-amino-4-methyl­pyridin-1-ium 2-hy­droxy-3,4-dioxo­cyclo­but-1-en-1-olate), C_6_H_9_N_2_^+^·C_4_HO_4_^−^, was obtained by the proton-transfer reaction between 2-amino-4-methyl­pyridine and squaric acid in aqueous solution: protonation occurs at the pyridine nitro­gen atom, while squaric acid is singly deprotonated. In the crystal, the cations and anions are linked by N—H⋯O, O—H⋯O, and C—H⋯O hydrogen bonds, forming infinite layers. These layers are consolidated by π–π stacking inter­actions, resulting in a columnar packing arrangement. Hirshfeld surface analysis reveals that O⋯H/H⋯O contacts dominate the inter­molecular inter­actions, consistent with the hydrogen-bonding network observed in the crystal structure.

## Abstract 2

The extended structure of the title salt features a network of N—H⋯O, O—H⋯O, and C—H⋯O hydrogen bonds, which generate infinite layers.

## 1. Chemical context

Proton-transfer mol­ecular salts arise when a Brønsted acid donates a proton to a Brønsted base, generating oppositely charged ions stabilized by charge-assisted hydrogen bonds and other non-covalent inter­actions (Aakeröy *et al.*, 2007 ▸). If proton transfer does not occur, a co-crystal may result in the solid state (Cruz-Cabeza *et al.*, 2022 ▸; Gilli *et al.*, 2002 ▸; Lemmerer *et al.*, 2015 ▸; Cruz-Cabeza, 2012 ▸). As to the outcome of a particular reaction, a simple qualitative approach is to consider the difference in p*K*_a_ values (Δp*K*_a_) between the conjugate acid of the base (BH^+^) and the acid (HA). If Δp*K*_a_ < 0, the system favors a co-crystal (all components remain neutral), while Δp*K*_a_ > 3 favors salt formation (Cruz-Cabeza, 2012 ▸; Cruz-Cabeza *et al.*, 2022 ▸). For inter­mediate Δp*K*_a_ values, however, the outcome is less predictable (Delori *et al.*, 2013 ▸). Since hydrogen-bonded systems derived from organic cations and anions often form stronger hydrogen bonds than their neutral counterparts (Bertolasi *et al.*, 2001 ▸), these systems have become increasingly important in crystal engineering and materials science because their structural frameworks and physicochemical properties can be finely tuned. Compared with their neutral precursors, proton-transfer salts often show greater solubility, stability, and functionality, which makes them attractive candidates for pharmaceuticals (Zhao *et al.*, 2020 ▸; Goswami *et al.*, 2025 ▸), optoelectronic materials (Huang *et al.*, 2022 ▸; K. K *et al.*, 2025 ▸; Sangtani *et al.*, 2017 ▸), and supra­molecular assemblies.

Squaric acid (3,4-di­hydroxy­cyclo­but-3-ene-1,2-dione, C_4_H_2_O_4_) and its derivatives have attracted significant attention in organic chemistry, materials science, and medicinal chemistry (Chasák *et al.*, 2021 ▸; Grus *et al.*, 2021 ▸, Laramie *et al.*, 2017 ▸). The inter­est in its structural chemistry arises from the planar, symmetrical, and strained nature of the squaric acid mol­ecule, which allows for diverse and robust hydrogen-bonding patterns in the solid state (Allen *et al.*, 2013 ▸; Gilli *et al.*, 2001 ▸). As a strong diprotic organic acid (p*K*_a1_ =1.2–1.7, p*K*_a2_ = 3.2–3.5; MacDonald, 1968 ▸), squaric acid readily forms proton-transfer compounds with nitro­gen bases, and numerous examples of such salts are recorded in the Cambridge Structural Database (Groom *et al.*, 2016 ▸). Upon deprotonation, squaric acid forms either the hydrogen squarate anion (Hsq^−^) or the squarate dianion (sq^2–^). All three species are nearly planar, featuring symmetric π-systems with extensive electronic delocalization with conjugated C=C and C=O bonds. This planarity, combined with their electronic structure, enables strong hydrogen-bonding inter­actions: while the squarate dianion acts exclusively as a hydrogen-bond acceptor, the parent acid and the mono-deprotonated hydrogen squarate ion can function as both donors and acceptors, making them versatile building blocks for supra­molecular architectures (Seidel & Kolev, 2024 ▸).

2-Amino-4-methyl-pyridine (2A4MP, C_6_H_8_N_2_) is a versatile pyridine-based heterocyclic compound in which the pyridine nitro­gen atom readily undergoes protonation, while the amine group donates electrons, facilitating the development of donor–acceptor (*D*–*A*) type systems. The ability of the pyridine N atom to accept protons from a wide range of organic acids, such as aromatic and aliphatic carb­oxy­lic acids, phenols, and related derivatives, makes it an excellent building block for the formation of stable mol­ecular salts. Proton-transfer salts of 2A4MP and related pyridinium derivatives have been widely explored for their nonlinear optical (NLO) properties including 2-amino-4-methylpyridinium 4-meth­oxybenzoate (Krishnakumar *et al.*, 2018 ▸), 2-amino-4-methyl­pyridinium 4-nitro­phenolate-4-nitro­phenol (Karuppusamy *et al.*, 2023 ▸; Thirupugalmani *et al.*, 2015 ▸), 2-amino-4-methyl­pyridinium benzilate (Madhankumar *et al.*, 2020 ▸) and others. As part of our studies in this area, the title proton-transfer mol­ecular salt, C_6_H_9_N_2_^+^·C_4_HO_4_^−^ (**I**), has been synthesized and its structural features are described here.
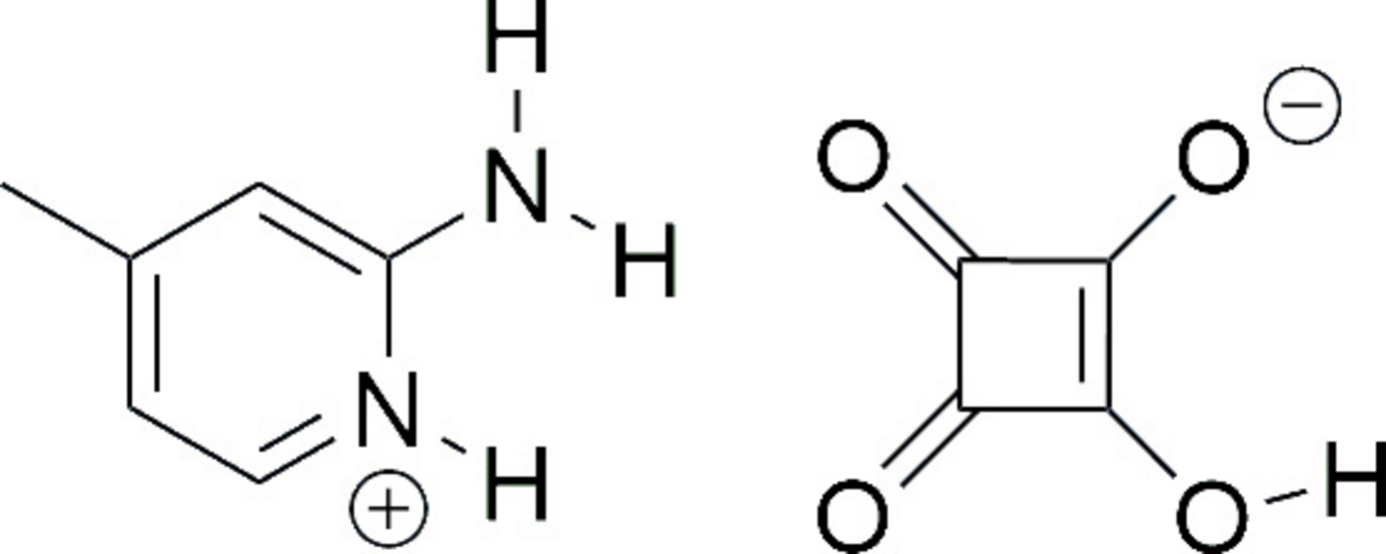


## 2. Structural commentary

**Figure 1:**
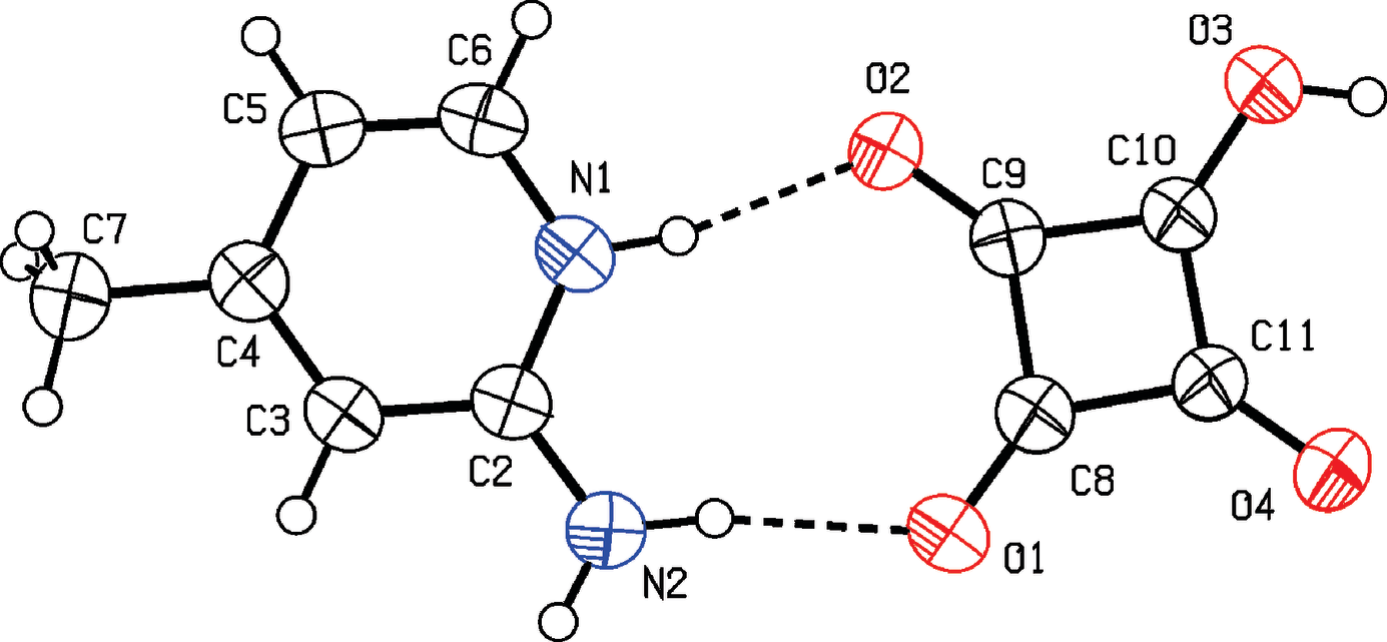
The mol­ecular structure of (**I**) with displacement ellipsoids drawn at the 50% probability level. Hydrogen bonds are indicated by dashed lines.

Salt (**I**) was obtained by proton transfer from squaric acid to 2-amino-4-methyl­pyridine in aqueous solution. The proton transfer observed in the title salt is consistent with the acidity constants of the components [p*K*_a_(squaric acid) ≃ 1.2–1.7; p*K*_a_(2-amino-4-methyl­pyridinium) ≃ 7.48], giving Δp*K*_a_ > 3, which favors salt formation rather than co-crystallization. Although Δp*K*_a_ relative to the second dissociation of squaric acid (p*K*_a_ ≃ 3.2–3.5) is also greater than 3, the formation of the fully deprotonated squarate dianion does not occur here. Whether squaric acid is mono- or fully deprotonated in the solid state depends on various factors such as stoichiometry, crystallization conditions and inter­molecular inter­actions, especially hydrogen bonds. In this case, the isolated crystalline product is the 1:1 hydrogen-squarate salt (Hsq^−^), which is consistent with the 1:1 stoichiometry and charge-assisted hydrogen bonding that favours the monoanion. Salt (**I**) crystallizes in the monoclinic system, space group *P*2_1_/*c*. The asymmetric unit contains one C_6_H_9_N_2_^+^ 2-amino-4-methyl­pyridin-1-ium cation and one C_4_HO_4_^−^ hydrogen squarate anion (Fig. 1 ▸) in which protonation occurs at the pyridine nitro­gen atom N1.

Both ions are nearly planar. The hydrogen squarate (Hsq^−^) anion deviates by less than 0.01 Å from planarity, and the cation shows a similar small deviation. The two planes are almost parallel, with a dihedral angle of 5.59 (12)° between the species in the asymmetric unit. Within the squarate ring, the C—C—C bond angles are close to 90°, while bond distances reveal partial bond localization: the shorter C—C bonds [C9—C10, 1.436 (2); C10—C11, 1.424 (2) Å] suggest double-bond character whereas the longer bonds [C8—C9, 1.484 (2); C8—C11, 1.479 (2) Å] resemble single bonds. Among the C—O bonds, one is short [C8—O1 = 1.228 (2) Å], while the others are elongated [C9—O2, 1.252 (2); C11—O4, 1.255 (2); C10—O3, 1.298 (2) Å]. The unusually long C10—O3 bond marks the main site of negative charge, which is delocalized over O2 and O4, consistent with resonance in the hydrogen squarate monoanion. Overall, the alternation of elongated and shortened C—C and C—O bonds highlights the extent of delocalization in the Hsq^−^ ion, in agreement with previous reports (Gołdyn *et al.*, 2022 ▸; Dega-Szafran *et al.*, 2012*c* ▸; 2013*a* ▸).

## 3. Supra­molecular features

**Figure 2:**
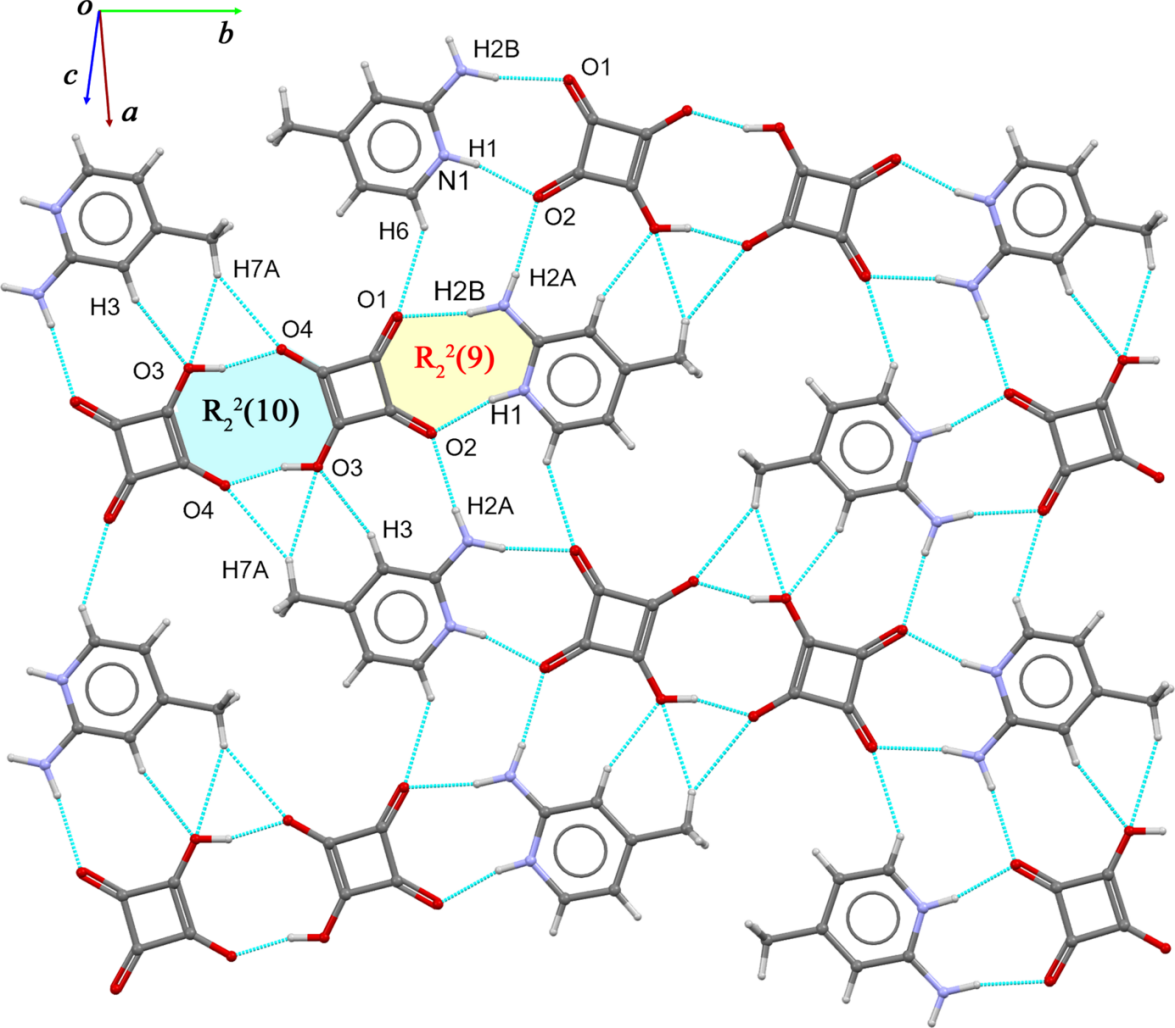
Part of the crystal structure of (**I**), showing the infinite layers formed through N—H⋯O, O—H⋯O, and C—H⋯O hydrogen bonds.

**Figure 3:**
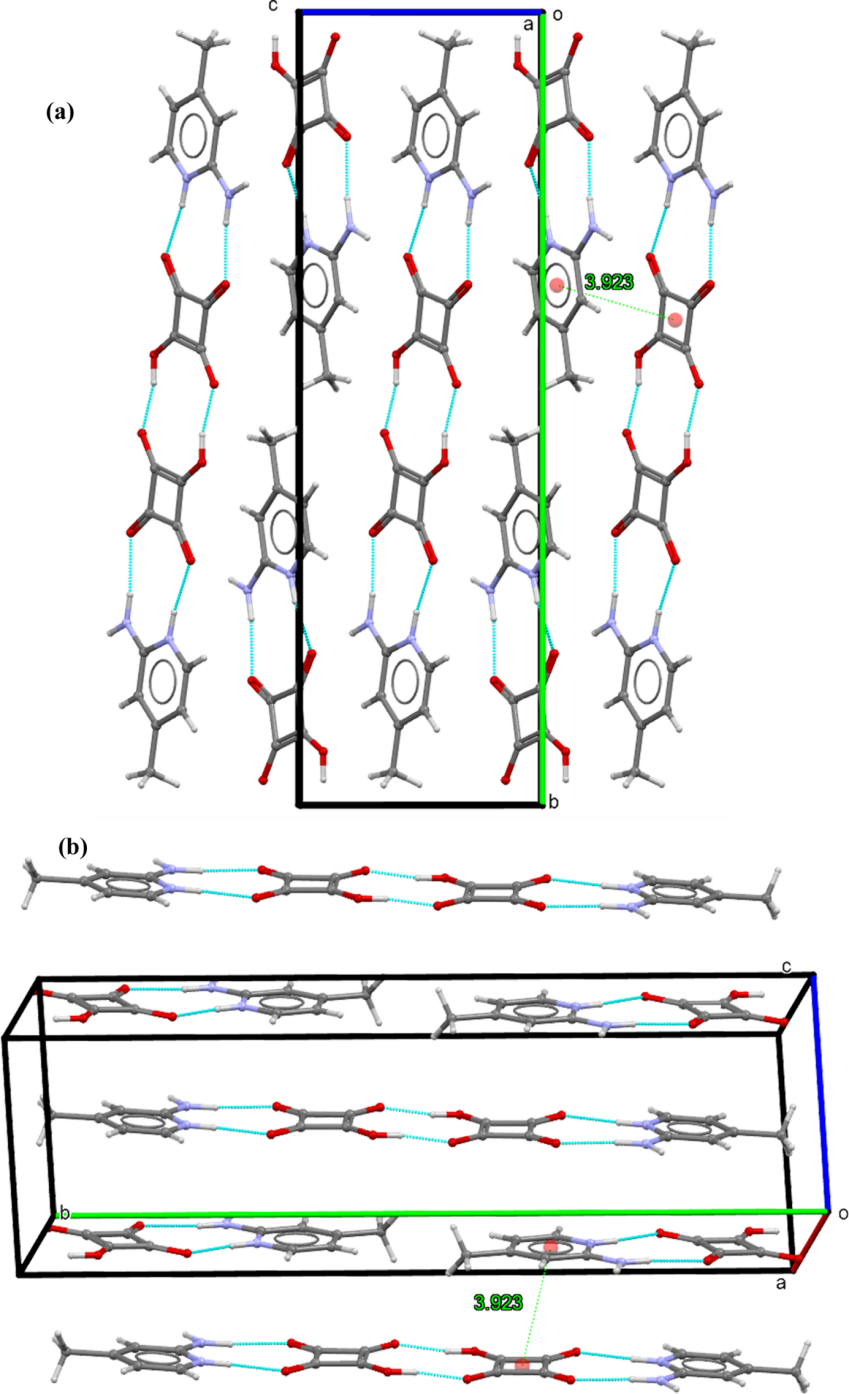
Overall crystal packing of (**I**), illustrating the columnar arrangement of the layers.

In the extended structure, all four oxygen atoms of the HSq^−^ anion, along with the amine group, the protonated pyridinium N atom and the hydroxyl hydrogen atom, act as hydrogen-bond donors and acceptors, giving rise to a network of N—H⋯O, O—H⋯O, and C—H⋯O hydrogen bonds within the crystal structure (Table 1 ▸). In the asymmetric unit, the cation and anion are connected through N1—H1⋯O2 and N2—H2*B*⋯O1 hydrogen bonds, forming an 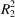
(9) motif. Two HSq^−^ anions generate a centrosymmetric dimer *via* pairwise O3—H3*A*⋯O4 links, which corresponds to an 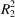
(10) motif. These HSq^−^ dimers are further linked by N2—H2*A*⋯O2 inter­actions, together with C—H⋯O contacts involving C3 and C6. Together, these inter­actions link the cations and anions into infinite layers propagating in the (10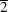) plane (Fig. 2 ▸). Adjacent layers are connected in the *z* direction through weak π–π stacking inter­actions between the squarate ring and the pyridine ring with a centroid–centroid distance of 3.9234 (13) Å (slippage = 2.096 Å); symmetry: *x*, *y*, 1 + *z*). Overall, these hydrogen-bonding and stacking inter­actions direct the assembly of cations and anions into a columnar arrangement, as shown in Fig. 3 ▸.

## 4. Hirshfeld surface analysis

*Crystal Explorer 21* (Turner *et al.*, 2017 ▸) was used to calculate the Hirshfeld surfaces of the cation and anion of the title salt and to generate two-dimensional fingerprint plots for the analysis and qu­anti­fication of various inter­molecular inter­actions in the crystal packing.

**Figure 4:**
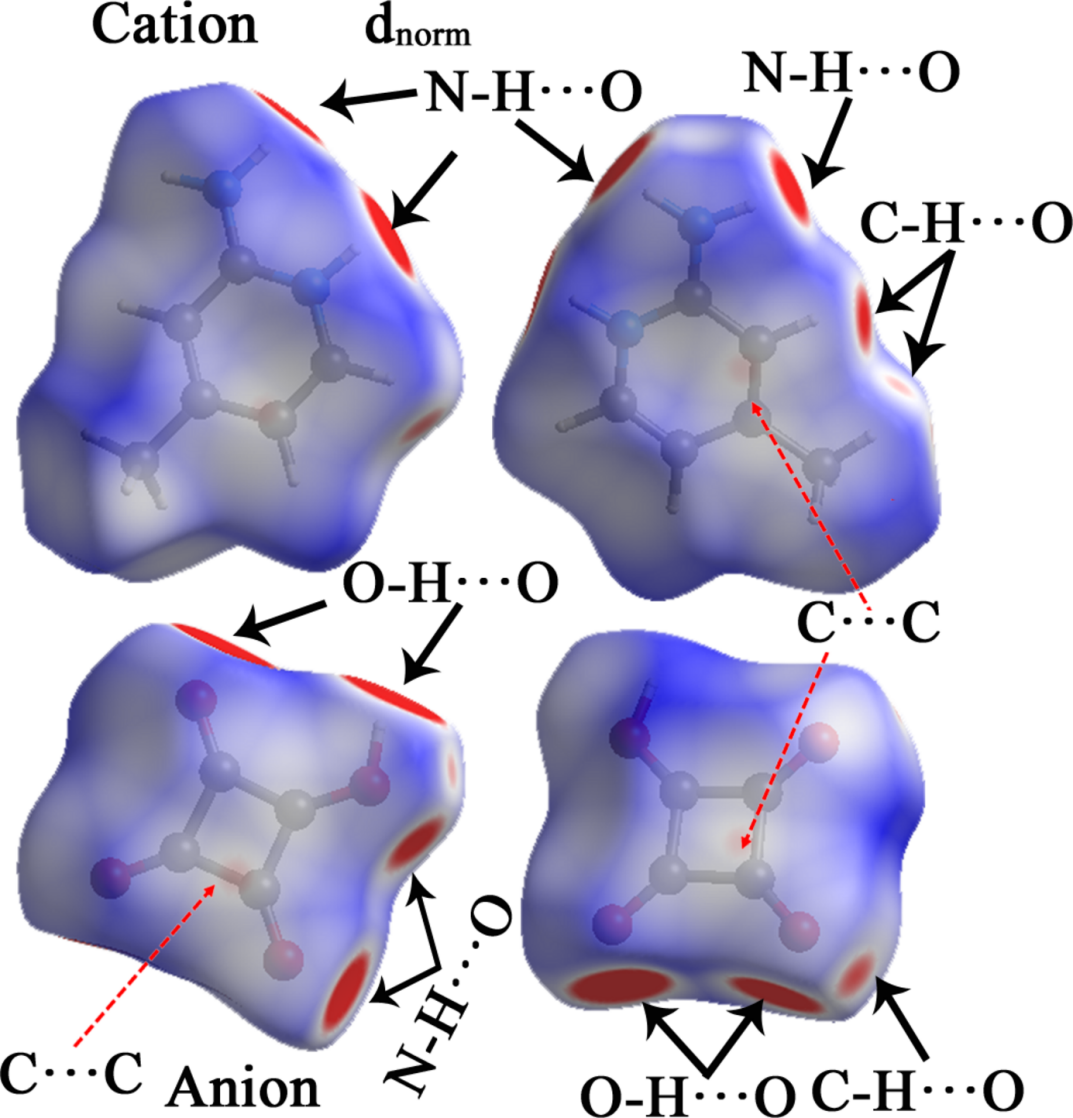
Two different orientations of the Hirshfeld surface of (**I**) mapped over *d*_norm_

The HS mapped over *d_norm_* within the range of −0.27 to 1.15 a.u., and two views (front and back) of the HS for both the cation and anion are shown in Fig. 4 ▸. Prominent bright-red spots on the HS confirm the presence of significant hydrogen-bonding inter­actions, specifically N—H⋯O and O—H⋯O inter­actions in the crystal structure. Weaker red spots appear near the carbon atoms in both the squarate ring (*Cg*1) and the pyridine ring (*Cg*2), which correspond to π–π stacking inter­actions.

**Figure 5:**
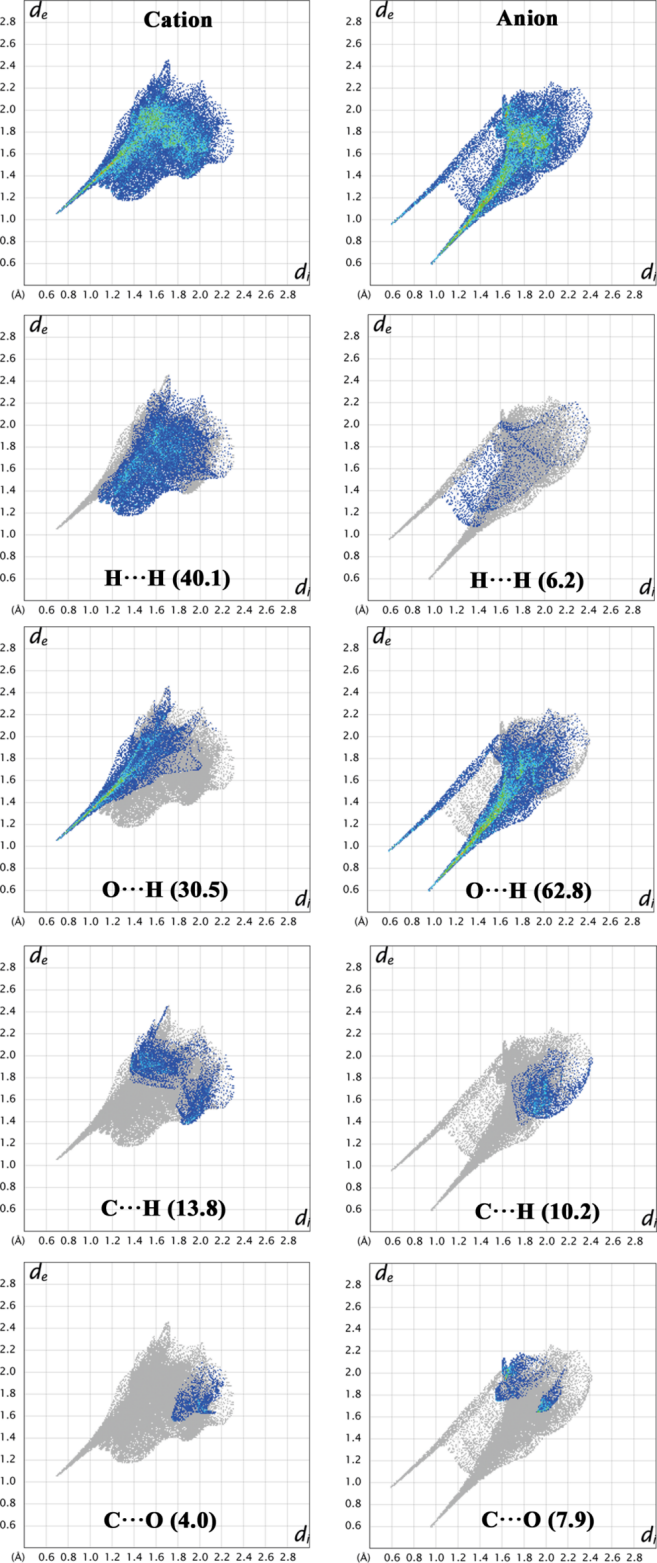
Two-dimensional fingerprint plots for the cation and anion in (**I**) showing the percentage contribution of the different contacts to the total Hirshfeld surface area.

The two-dimensional fingerprint and decomposed plots for the individual components (cation and anion) are presented in Fig. 5 ▸. H⋯H contacts contribute 40.1% to the cation surface, representing the dominant inter­action in its crystal packing. In contrast, for the anion, these contacts account for only 6.2% of the surface, ranking as the fourth major contributor to stabilization. The corresponding FP plots reveal a deep, asymmetric broad spike around *d*_e_ + *d*_i_ > 2.4 Å (where *d*_e_ and *d*_i_ denote the distances from the Hirshfeld surface to the nearest nucleus outside and inside the surface, respectively) for the cation, whereas in the anion, these contacts appear as weak and well-separated spots. These distinct shapes suggest markedly different hydrogen environments and inter­action patterns between the cationic and anionic units. The next major inter­actions are O⋯H/H⋯O contacts, contributing 30.5% and 62.8% for the cation and anion, respectively, indicating that these contacts play a dominant role in the anion. In the cation, this contact appears as a single sharp spike at *d*_e_ + *d*_i_ = 1.6 Å, while in the anion, two distinct spikes are observed at the same distance, reflecting stronger and more varied hydrogen-bonding environments. C⋯H/H⋯C inter­actions contribute 13.8% and 10.2% to the cation and anion surfaces, respectively. Other minor contributions include N⋯H/H⋯N (5.5%), C⋯C (4.8%), and C⋯O/O⋯C (4.0%) for the cation, and C⋯O/O⋯C (7.9%), O⋯O (5.8%), and N⋯O/O⋯N (1.0%) for the anion. Overall, the FP analysis reveals that both the cation and anion are predominantly stabilized through C/N/O—H⋯O-type hydrogen-bonding inter­actions, although their spatial distribution and intensity differ significantly.

## 5. Database survey

A search of the Cambridge Structural Database (CSD, Version 6.00, updates of April 2025 and August 2025; (Groom *et al.*, 2016 ▸) using Conquest (Bruno *et al.*, 2002 ▸) identified 52 entries containing the neutral squaric acid mol­ecule. Among these, 14 hits correspond to purely neutral squaric acid, while eight represent co-crystals formed with various organic bases, including *N*-methyl­piperidine betaine (CSD refcode CAPKUB; Dega-Szafran *et al.*, 2012*b* ▸), *N*-ethyl­piperidine betaine (CILQAR; Dega-Szafran *et al.*, 2013*a* ▸), pyridinium-2-carboxyl­ate (HETSEI; Gołdyn *et al.*, 2022 ▸), 2-(quinuclidinium)propionate (DIMSUP; Dega-Szafran *et al.*, 2013*b* ▸), trigonelline (PAKNUM; Dega-Szafran *et al.*, 2012**a* ▸)*, pyrazine­carboxamide (PAQNOM; Korkmaz *et al.*, 2011 ▸), urea (QIRKAD; Sabareesh *et al.*, 2001 ▸) and glycine (SIZKIX01; Tyagi *et al.*, 2016 ▸).

For hydrogen squarate species, 193 structures were found, comprising 15 metal complexes, with the remaining being mono-deprotonated salts involving a variety of aliphatic primary amines, amino acids, and nitro­gen containing heterocycles such as pyridine, bi­pyridine and related compounds. Furthermore, 104 entries were found containing the squarate dianion.

A separate CSD search for the 2-amino-4-methyl­pyridin-1-ium cation revealed 63 entries, most of which arise from the reaction of 2-amino-4-methyl­pyridine with various aliphatic or aromatic carb­oxy­lic acid and phenolic co-formers.

## 6. Synthesis and crystallization

Squaric acid (2.28 g, 0.0199 mmol) and 2-amino-4-methyl­pyridine (2.162 g, 0.0199 mmol) were dissolved in 25 ml of double-distilled water and stirred at room temperature (298 K) for 4 h. The reaction mixture was then filtered and allowed to evaporate slowly at room temperature, yielding yellow plates of (**I**) suitable for X-ray diffraction analysis.

## 7. Refinement

Crystal data, data collection and structure refinement details are summarized in Table 2 ▸. The N-bound H atoms were located in a difference-Fourier map and refined with isotropic displacement parameters. All C-bound H atoms were included in calculated positions and treated as riding atoms with C–H = 0.93–0.98 Å and *U*_iso_(H) = 1.2*U*_eq_(C).

## Supplementary Material

Crystal structure: contains datablock(s) I. DOI: 10.1107/S205698902501045X/hb8170sup1.cif

Structure factors: contains datablock(s) I. DOI: 10.1107/S205698902501045X/hb8170Isup3.hkl

Supporting information file. DOI: 10.1107/S205698902501045X/hb8170Isup3.cml

CCDC reference: 2504530

Additional supporting information:  crystallographic information; 3D view; checkCIF report

## Figures and Tables

**Table 1:** Hydrogen-bond geometry (Å, °)

*D*—H⋯*A*	*D*—H	H⋯*A*	*D*⋯*A*	*D*—H⋯*A*
N1—H1⋯O2	0.88 (2)	1.87 (2)	2.734 (2)	167 (2)
N2—H2*A*⋯O2^i^	0.87 (2)	2.16 (2)	3.025 (2)	175 (2)
N2—H2*B*⋯O1	0.91 (2)	1.95 (2)	2.856 (2)	170 (2)
O3—H3*A*⋯O4^ii^	0.87 (2)	1.67 (2)	2.5145 (19)	163 (3)
C3—H3⋯O3^i^	0.93	2.34	3.230 (2)	159
C6—H6⋯O1^iii^	0.93	2.49	3.284 (2)	143

Symmetry codes: (i) 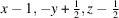; (ii) 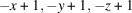; (iii) 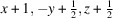.

**Table 2:** Experimental details

Crystal data	
Chemical formula	C_6_H_9_N_2_^+^·C_4_HO_4_^−^
*M*_r_	222.20
Crystal system, space group	Monoclinic, *P*2_1_/*c*
Temperature (K)	305
*a*, *b*, *c* (Å)	5.3441 (3), 24.9059 (12), 7.6505 (4)
β (°)	95.597 (3)
*V* (Å^3^)	1013.42 (9)
*Z*	4
Radiation type	Cu *K*α
μ (mm^−1^)	0.97
Crystal size (mm)	0.32 × 0.13 × 0.04

Data collection	
Diffractometer	Bruker D8 Venture Diffractometer
Absorption correction	Multi-scan (*SADABS*; Krause *et al.*, 2015 ▸)
*T*_min_, *T*_max_	0.731, 1.000
No. of measured, independent and observed [*I* > 2σ(*I*)] reflections	20467, 1860, 1602
*R*_int_	0.063
(sin θ/λ)_max_ (Å^−1^)	0.602

Refinement	
*R*[*F*^2^ > 2σ(*F*^2^)], *wR*(*F*^2^), *S*	0.048, 0.133, 1.05
No. of reflections	1860
No. of parameters	159
No. of restraints	4
H-atom treatment	H atoms treated by a mixture of independent and constrained refinement
Δρ_max_, Δρ_min_ (e Å^−3^)	0.21, −0.16

Computer programs: *APEX4*, *SAINT* and *XPREP* (Bruker, 2021 ▸), *SHELXT2018/2* (Sheldrick, 2015*a* ▸) *SHELXL2019/2* (Sheldrick, 2015*b* ▸), *ORTEP-3 for Windows* (Farrugia, 2012 ▸) and * Mercury* (Macrae *et al.*, 2020 ▸).

## supplementary crystallographic information

### 2-amino-4-methylpyridin-1-ium 2-hydroxy-3,4-dioxocyclobut-1-en-1-olate Crystal data

**Table:**

C_6_H_9_N_2_^+^·C_4_HO_4_^−^	*F*(000) = 464
*M**_r_* = 222.20	*D*_x_ = 1.456 Mg m^−^^3^
Monoclinic, *P*2_1_/*c*	Cu *K*α radiation, λ = 1.54178 Å
*a* = 5.3441 (3) Å	Cell parameters from 9939 reflections
*b* = 24.9059 (12) Å	θ = 3.6–68.2°
*c* = 7.6505 (4) Å	µ = 0.97 mm^−^^1^
β = 95.597 (3)°	*T* = 305 K
*V* = 1013.42 (9) Å^3^	Plate, yellow
*Z* = 4	0.32 × 0.13 × 0.04 mm

### 2-amino-4-methylpyridin-1-ium 2-hydroxy-3,4-dioxocyclobut-1-en-1-olate Data collection

**Table:**

Bruker D8 Venture Diffractometer	1602 reflections with *I* > 2σ(*I*)
Radiation source: micro focus sealed tube	*R*_int_ = 0.063
φ and ω scans	θ_max_ = 68.2°, θ_min_ = 3.6°
Absorption correction: multi-scan (SADABS; Krause *et al.*, 2015)	*h* = −6→6
*T*_min_ = 0.731, *T*_max_ = 1.000	*k* = −30→29
20467 measured reflections	*l* = −9→9
1860 independent reflections	

### 2-amino-4-methylpyridin-1-ium 2-hydroxy-3,4-dioxocyclobut-1-en-1-olate Refinement

**Table:**

Refinement on *F*^2^	Hydrogen site location: mixed
Least-squares matrix: full	H atoms treated by a mixture of independent and constrained refinement
*R*[*F*^2^ > 2σ(*F*^2^)] = 0.048	*w* = 1/[σ^2^(*F*_o_^2^) + (0.0687*P*)^2^ + 0.3185*P*] where *P* = (*F*_o_^2^ + 2*F*_c_^2^)/3
*wR*(*F*^2^) = 0.133	(Δ/σ)_max_ < 0.001
*S* = 1.05	Δρ_max_ = 0.21 e Å^−^^3^
1860 reflections	Δρ_min_ = −0.16 e Å^−^^3^
159 parameters	Extinction correction: *SHELXL2019/2* (Sheldrick, 2015b), Fc^*^=kFc[1+0.001xFc^2^λ^3^/sin(2θ)]^-1/4^
4 restraints	Extinction coefficient: 0.0087 (17)

### 2-amino-4-methylpyridin-1-ium 2-hydroxy-3,4-dioxocyclobut-1-en-1-olate Special details

**Table:**

Geometry. All esds (except the esd in the dihedral angle between two l.s. planes) are estimated using the full covariance matrix. The cell esds are taken into account individually in the estimation of esds in distances, angles and torsion angles; correlations between esds in cell parameters are only used when they are defined by crystal symmetry. An approximate (isotropic) treatment of cell esds is used for estimating esds involving l.s. planes.

### 2-amino-4-methylpyridin-1-ium 2-hydroxy-3,4-dioxocyclobut-1-en-1-olate Fractional atomic coordinates and isotropic or equivalent isotropic displacement parameters (Å^2^)

**Table:**

	*x*	*y*	*z*	*U*_iso_*/*U*_eq_	
C8	0.2327 (4)	0.36789 (7)	0.3849 (3)	0.0511 (5)	
C9	0.4649 (3)	0.35258 (7)	0.4961 (2)	0.0484 (4)	
C10	0.5292 (3)	0.40844 (7)	0.5131 (2)	0.0503 (5)	
C11	0.3083 (3)	0.42479 (7)	0.4074 (2)	0.0508 (5)	
C2	0.1668 (3)	0.19296 (7)	0.3631 (2)	0.0490 (5)	
C3	0.1295 (3)	0.13741 (7)	0.3398 (3)	0.0513 (5)	
H3	−0.014079	0.125298	0.272925	0.062*	
C4	0.2983 (3)	0.10099 (8)	0.4125 (2)	0.0516 (5)	
C5	0.5147 (4)	0.12001 (8)	0.5150 (3)	0.0562 (5)	
H5	0.633864	0.096078	0.566124	0.067*	
C6	0.5456 (3)	0.17340 (8)	0.5375 (3)	0.0555 (5)	
H6	0.687478	0.186086	0.604995	0.067*	
C7	0.2546 (4)	0.04188 (8)	0.3877 (3)	0.0694 (6)	
H7A	0.114532	0.036192	0.301245	0.104*	
H7B	0.219224	0.026088	0.497082	0.104*	
H7C	0.402203	0.025529	0.348913	0.104*	
N1	0.3744 (3)	0.20903 (6)	0.4638 (2)	0.0515 (4)	
H1	0.412 (4)	0.2429 (7)	0.484 (3)	0.062*	
N2	0.0057 (3)	0.22921 (7)	0.2916 (3)	0.0667 (5)	
H2A	−0.121 (4)	0.2164 (10)	0.225 (3)	0.080*	
H2B	0.038 (5)	0.2650 (7)	0.306 (3)	0.080*	
O1	0.0553 (3)	0.34331 (5)	0.3083 (2)	0.0667 (5)	
O2	0.5578 (2)	0.30894 (5)	0.55156 (19)	0.0590 (4)	
O3	0.7207 (3)	0.43223 (6)	0.5973 (2)	0.0714 (5)	
H3A	0.719 (6)	0.4672 (7)	0.598 (4)	0.107*	
O4	0.2147 (3)	0.46871 (5)	0.3536 (2)	0.0708 (5)	

### 2-amino-4-methylpyridin-1-ium 2-hydroxy-3,4-dioxocyclobut-1-en-1-olate Atomic displacement parameters (Å^2^)

**Table:**

	*U*^11^	*U*^22^	*U*^33^	*U*^12^	*U*^13^	*U*^23^
C8	0.0517 (10)	0.0445 (9)	0.0547 (10)	−0.0023 (8)	−0.0068 (8)	−0.0002 (8)
C9	0.0495 (9)	0.0401 (9)	0.0534 (10)	−0.0016 (7)	−0.0053 (8)	0.0007 (7)
C10	0.0521 (10)	0.0407 (9)	0.0547 (10)	−0.0042 (7)	−0.0117 (8)	0.0006 (7)
C11	0.0550 (10)	0.0398 (9)	0.0544 (10)	−0.0006 (7)	−0.0117 (8)	0.0034 (7)
C2	0.0439 (9)	0.0463 (10)	0.0547 (10)	−0.0023 (7)	−0.0055 (8)	−0.0001 (8)
C3	0.0468 (9)	0.0449 (10)	0.0594 (11)	−0.0055 (7)	−0.0097 (8)	−0.0023 (8)
C4	0.0505 (10)	0.0465 (10)	0.0562 (10)	−0.0037 (8)	−0.0033 (8)	0.0015 (8)
C5	0.0493 (10)	0.0517 (11)	0.0648 (12)	0.0020 (8)	−0.0094 (9)	0.0043 (9)
C6	0.0439 (9)	0.0581 (11)	0.0614 (11)	−0.0050 (8)	−0.0105 (8)	0.0003 (9)
C7	0.0701 (13)	0.0455 (11)	0.0877 (15)	−0.0022 (9)	−0.0168 (11)	0.0056 (10)
N1	0.0473 (8)	0.0451 (8)	0.0596 (9)	−0.0060 (6)	−0.0082 (7)	−0.0028 (7)
N2	0.0586 (10)	0.0449 (9)	0.0902 (13)	0.0002 (8)	−0.0249 (9)	−0.0001 (9)
O1	0.0583 (8)	0.0490 (8)	0.0864 (10)	−0.0072 (6)	−0.0246 (7)	−0.0013 (7)
O2	0.0577 (8)	0.0376 (7)	0.0768 (9)	0.0006 (5)	−0.0183 (6)	0.0037 (6)
O3	0.0683 (9)	0.0438 (8)	0.0935 (11)	−0.0083 (6)	−0.0349 (8)	0.0030 (7)
O4	0.0780 (10)	0.0408 (7)	0.0861 (10)	0.0021 (6)	−0.0296 (8)	0.0071 (7)

### 2-amino-4-methylpyridin-1-ium 2-hydroxy-3,4-dioxocyclobut-1-en-1-olate Geometric parameters (Å, º)

**Table:**

C8—O1	1.228 (2)	C3—C4	1.359 (3)
C8—C11	1.479 (2)	C4—C5	1.414 (3)
C8—C9	1.484 (2)	C4—C7	1.500 (3)
C9—O2	1.252 (2)	C5—C6	1.349 (3)
C9—C10	1.436 (2)	C5—H5	0.9300
C10—O3	1.298 (2)	C6—N1	1.357 (2)
C10—C11	1.424 (2)	C6—H6	0.9300
C11—O4	1.255 (2)	C7—H7A	0.9600
C2—N2	1.328 (2)	C7—H7B	0.9600
C2—N1	1.348 (2)	C7—H7C	0.9600
C2—C3	1.407 (2)	N1—H1	0.876 (16)
			
O1—C8—C11	136.27 (18)	C5—C4—C7	120.50 (18)
O1—C8—C9	135.13 (17)	C6—C5—C4	118.95 (18)
C11—C8—C9	88.61 (14)	C6—C5—H5	120.5
O2—C9—C10	136.63 (17)	C4—C5—H5	120.5
O2—C9—C8	134.36 (16)	C5—C6—N1	121.49 (17)
C10—C9—C8	89.00 (14)	C5—C6—H6	119.3
O3—C10—C11	136.15 (17)	N1—C6—H6	119.3
O3—C10—C9	131.12 (17)	C4—C7—H7A	109.5
C11—C10—C9	92.73 (14)	C4—C7—H7B	109.5
O4—C11—C10	135.83 (18)	H7A—C7—H7B	109.5
O4—C11—C8	134.51 (17)	C4—C7—H7C	109.5
C10—C11—C8	89.66 (14)	H7A—C7—H7C	109.5
N2—C2—N1	119.86 (17)	H7B—C7—H7C	109.5
N2—C2—C3	122.58 (17)	C2—N1—C6	121.82 (17)
N1—C2—C3	117.57 (16)	C2—N1—H1	123.2 (16)
C4—C3—C2	121.62 (17)	C6—N1—H1	114.9 (16)
C4—C3—H3	119.2	C2—N2—H2A	115.3 (17)
C2—C3—H3	119.2	C2—N2—H2B	120.4 (17)
C3—C4—C5	118.53 (18)	H2A—N2—H2B	124 (2)
C3—C4—C7	120.96 (17)	C10—O3—H3A	117 (2)
			
O1—C8—C9—O2	−1.0 (4)	C9—C8—C11—O4	179.8 (2)
C11—C8—C9—O2	178.9 (2)	O1—C8—C11—C10	180.0 (3)
O1—C8—C9—C10	−180.0 (2)	C9—C8—C11—C10	0.12 (15)
C11—C8—C9—C10	−0.12 (15)	N2—C2—C3—C4	179.3 (2)
O2—C9—C10—O3	0.7 (4)	N1—C2—C3—C4	−1.3 (3)
C8—C9—C10—O3	179.7 (2)	C2—C3—C4—C5	0.5 (3)
O2—C9—C10—C11	−178.8 (2)	C2—C3—C4—C7	179.5 (2)
C8—C9—C10—C11	0.13 (15)	C3—C4—C5—C6	0.2 (3)
O3—C10—C11—O4	0.7 (4)	C7—C4—C5—C6	−178.7 (2)
C9—C10—C11—O4	−179.8 (2)	C4—C5—C6—N1	−0.1 (3)
O3—C10—C11—C8	−179.6 (3)	N2—C2—N1—C6	−179.1 (2)
C9—C10—C11—C8	−0.13 (15)	C3—C2—N1—C6	1.5 (3)
O1—C8—C11—O4	−0.4 (4)	C5—C6—N1—C2	−0.8 (3)

### 2-amino-4-methylpyridin-1-ium 2-hydroxy-3,4-dioxocyclobut-1-en-1-olate Hydrogen-bond geometry (Å, º)

**Table:**

*D*—H···*A*	*D*—H	H···*A*	*D*···*A*	*D*—H···*A*
N1—H1···O2	0.88 (2)	1.87 (2)	2.734 (2)	167 (2)
N2—H2*A*···O2^i^	0.87 (2)	2.16 (2)	3.025 (2)	175 (2)
N2—H2*B*···O1	0.91 (2)	1.95 (2)	2.856 (2)	170 (2)
O3—H3*A*···O4^ii^	0.87 (2)	1.67 (2)	2.5145 (19)	163 (3)
C3—H3···O3^i^	0.93	2.34	3.230 (2)	159
C6—H6···O1^iii^	0.93	2.49	3.284 (2)	143

Symmetry codes: (i) *x*−1, −*y*+1/2, *z*−1/2; (ii) −*x*+1, −*y*+1, −*z*+1; (iii) *x*+1, −*y*+1/2, *z*+1/2.
